# Wood’s Quarterly Retrospect of American and Foreign Practical Medicine and Surgery

**Published:** 1847-12

**Authors:** 


ARTICLE XVI.

*Wood's Quarterly Retrospect of American and Foreign Practical Medicine and Surgery.* p. 64. 8vo. New York. Richard & Geo. S. Wood. (In exchange.)

The first number of this work was received prior to the publication of our last number, but too late to receive a notice in its proper place.

It is on the plan of Braithwait's and Ranking's works, which we have already favorably noticed, but is to be fuller in reference to what appears each quarter in American medical literature.

It is a work which, from its character, being a synopsis of all that transpires in the way of improvements and discoveries in the profession generally, must be highly interesting, and as it is afforded at the low price of one dollar per annum, will probably have, as it should, a very extensive circulation. E.

